# Acculturation and obesity among migrant populations in high income countries – a systematic review

**DOI:** 10.1186/1471-2458-13-458

**Published:** 2013-05-10

**Authors:** Maryam Delavari, Anders Larrabee Sønderlund, Boyd Swinburn, David Mellor, Andre Renzaho

**Affiliations:** 1 WHO Collaborating Centre for Obesity Prevention, Department of Health, Deakin University, Melbourne, Australia; 2 School of Psychology, College of Life and Environmental Sciences, University of Exeter, Exeter, UK; 3 School of Population Health, University of Auckland, Auckland, New Zealand; 4 School of Psychology, Faculty of health, Deakin University, Melbourne, Australia; 5 Global Health and Society Unit, School of Public Health and Preventive Medicine, Monash University; and Centre for International Health, Burnet Institute, Melbourne, Australia

**Keywords:** Acculturation, Acculturation scale, Immigration, BMI, Diet

## Abstract

**Background:**

There is evidence to suggest that immigrant populations from low or medium-income countries to high income countries show a significant change in obesogenic behaviors in the host society, and that these changes are associated with acculturation. However, the results of studies vary depending on how acculturation is measured. The objective of this study is to systematically review the evidence on the relationship between acculturation - as measured with a standardized acculturation scale - and overweight/obesity among adult migrants from low/middle countries to high income countries.

**Methods:**

A systematic review of relevant studies was undertaken using six EBSCOhost databases and following the Centre for Reviews and Dissemination’s Guidance for Undertaking Reviews in Health Care.

**Results:**

The initial search identified 1135 potentially relevant publications, of which only nine studies met the selection criteria. All of the studies were from the US with migrant populations from eight different countries. Six studies employed bi-directional acculturation scales and three used uni-directional scales. Six studies indicated positive general associations between higher acculturation and body mass index (BMI), and three studies reported that higher acculturation was associated with lower BMI, as mainly among women.

**Conclusion:**

Despite the small number of studies, a number of potential explanatory hypotheses were developed for these emerging patterns. The ‘Healthy Migrant Effect’ may diminish with greater acculturation as the host culture potentially promotes more unhealthy weight gain than heritage cultures. This appears particularly so for men and a rapid form of nutrition transition represents a likely contributor. The inconsistent results observed for women may be due to the interplay of cultural influences on body image, food choices and physical activity. That is, the Western ideal of a slim female body and higher values placed on physical activity and fitness may counteract the obesogenic food environment for female migrants.

## Background

In 2008, over 1.5 billion adults around the world were classified as being overweight (BMI ≥ 25) [1], with an increase in prevalence from 4.8% to 9.8% since 1980 [2].

The determinants of overweight and obesity are manifold, with certain subgroups of the population seemingly more at risk [3,4]. In particular, people from low- to medium-income countries who have migrated to and reside in high-income countries^1^, appear to be more susceptible to overweight and obesity than their local counterparts [5,6]. This is the case even though newly arrived migrants tend to be healthier than the host population - a phenomenon called *the healthy migrant effect*[7,8]. Reasons for the healthy migrant effect include the positive selection bias that the formal migration process imposes, such as the rigorous health checks required pre-migration [9-15].

The unhealthy weight gain among migrants appears to increase significantly over 10–15 years post migration, by which time migrants’ overweight and obesity rates approach or overtake those of the host population [16-18]. This change, however, is not uniform across all migrant groups, and may vary as a result of actual differences between groups such as ethnicity, gender, age at the time of migration, and period of residency in the new country [19-21]. For example, a health report from Canada found that black immigrant women were more likely to be overweight compared with white immigrant women, regardless of when they immigrated [22]. Similarly, data from a health survey in South Australia demonstrated significant differences between migrants from different ethnic groups in the prevalence of overweight and obesity [23]. Among US- and Australian-based migrant groups, people born in Italy, Greece or Cyprus, former Yugoslavia, Germany, the Netherlands, Poland and other Eastern European countries recorded a higher likelihood of overweight and obesity than people born in Malaysia, Vietnam or Cambodia, the Philippines, or China. Additionally, a number of studies found significant positive correlations between length of residency in the host country and weight gain [20,24,25]. Finally, other research has established an inverse relationship between migrants’ age at the time of immigration and overweight/obesity [20,26].

Thus, when examining the relationship between migration and health-related factors such as obesity, there appears to be a wide variety of factors at play, including the healthy migrant effect, length of residence in host country, gender, country of origin, and age.

### Acculturation & enculturation

The underlying dynamics of the complex relationship between migration and migrant obesity may be further clarified with a theoretical perspective which takes into account the forces of acculturation and enculturation [27-31]. As indicated above, the majority of people who migrate from low-income to high-income countries eventually adopt obesogenic behaviours, experience weight gain, and record higher body weights than their local counterparts. This change occurs as their immersion into the host culture increases over time [5,6,24] and is indicative of the broader and more complex process of *acculturation* – that is, the gradual exchange between immigrants’ original attitudes and behaviour and those of the host culture [32]. This phenomenon has consistently been associated with negative consequences for migrants, including added stressors, fewer coping resources, and poorer mental health [33].

In contrast some migrants may avoid these adverse effects of immigration, by assimilating less to the host culture and retaining more of their traditional heritage. This is termed *enculturation*[34], and often appears to be beneficial to migrants in terms of social and health effects. In particular, this phenomenon is commonly associated with the maintenance of several protective factors, including social/familial support, shared sense of ethnic identity, and continued practice of traditional values [35].

Thus, acculturation and enculturation are intertwined and representative of important psychosocial factors in the migration process - not least in terms of several health-risks. Indeed it would appear that acculturation is generally associated with adverse health-effects, and enculturation the opposite. Hence, in order to gain a clearer understanding of the impact of migration on migrant health, these two factors must be included in the analysis.

While past research has examined the relationship betweenx migrant health and acculturation, most studies have employed surrogate measures – such as for example length of residency in the host culture, place of birth or preferred language used by immigrants [36,37] – rather than using standardised acculturation scales. Due to such lack of reliable and consistent methods for quantifying the degree of acculturation, comparative study and meta-analyses have been difficult, if not impossible, leaving a considerable gap in the literature on this topic. Furthermore, the fact that only few studies have taken enculturation into account, has meant that the research in this field has generally failed to separate the differential impact of acculturation and enculturation [38,39]. This shortcoming represents another limitation of research in this area.

The two most commonly used standardised models of acculturation are uni-directional models (UDM) and bi-directional models (BDM). UDMs are based on the assumption that the maintenance of the original migrant heritage is not possible among those immigrants who become involved in the mainstream host society. Thus, acculturation is inevitably accompanied by a weakening of identification with heritage, and can be represented as a linear change over time.

Similarly, BDMs suggest that the relationship with both original heritage and the new host culture plays an important role in the process of acculturation [36]. This model allows for interaction effects of the original and the new cultures by assessing an individual’s cultural identity and participation in the larger society [40].

In summary, the use of acculturation scales may help to more accurately explain the complex relationship between migration and obesity**,** and further may do so in a more standardised, quantifiable and comparable fashion than past research. The aim of this study is therefore:

To systematically review the evidence on the relationship between acculturation - as measured with a standardized acculturation scale (i.e. UDM or BDM) - and overweight/obesity among adult migrants from low/middle countries to high income countries.

## Methods

### Protocol

This review was conducted according to the Preferred Reporting Items for Systematic Reviews and Meta-Analyses (PRISMA). This system has been developed to provide clear, practical and systematic guidance in researching and writing systematic reviews.

### Information sources

A comprehensive search of the following databases was conducted during May 2011: Academic Search Complete (EBSCOhost); Academic Search Premier (EBSCOhost); CINAHL with Full Text (EBSCOhost); Global Health (EBSCOhost); MEDLINE with Full Text (EBSCOhost); and PsycINFO (EBSCOhost).

### Search strategy & study selection process

The search terms used were based on MeSH keywords for ‘obesity’, ‘enculturation’ and ‘acculturation’. Searches were conducted by inserting the following terms simultaneously into the EBSCOhost database. Within term groups (that is Obesity terms and Acculturation terms) search terms were divided with ‘or’, while the two term groups were connected with ‘and’. The search strategy including databases, keywords, and limits is defined in Table 1.

**Table 1:** Search strategy including databases, keywords, and limits

Databases searched	Academic Search Complete (EBSCOhost); Academic Search Premier (EBSCOhost); CINAHL with Full Text (EBSCOhost); Global Health (EBSCOhost); MEDLINE with Full Text (EBSCOhost); and PsycINFO (EBSCOhost).
Key Words	MeSH keywords: (Obes* OR Overweight OR Weight gain OR Bodyweight OR Body mass index OR Waist-hip ratio) AND (Enculturat* OR Acculturat* OR assimilat* OR Integrat* OR Cultural change OR Biculturalism OR Cultural integration OR Culture diffusion OR Cultural adaptation OR Cultural shift Or Social integration) Additional keywords: (Obesity OR overweight person OR overweight OR weight gain OR body weight OR body mass index OR waist-hip ratio) AND (Acculturation OR culture change OR assimilation OR integration OR social integration OR biculturalism OR cultural integration OR culture diffusion OR cultural change OR cultural adaptation OR cultural shift).
Inclusion criteria	i. The study was published in a peer-reviewed scientific journal. ii. The study used an acculturation scale (UDM or BDM) to measure acculturation. iii. The study did not rely on surrogate measures of acculturation (e.g. length of stay in host country, generations (1st vs. 2nd), or nativity/birthplace). iv. The population under study comprised migrants and/or refugees from low- or medium-income countries living in high-income countries. v. The populations under study comprised adults over 18 years of age. The paper was published between January 1990 and May 2011.

### Validity & quality assessment

Articles obtained in the search were appraised in terms of relevance, quality and validity in three rounds. In the first round, articles were included or excluded based on their title. In the second round, articles were retained or excluded after reviewing their abstracts. In the third round, the full-text version of each of the remaining articles was obtained [41] and reviewed separately by two researchers. To determine the quality of the studies reviewed, quality assessment tools were utilised. The strength of the quantitative studies was ranked using the *Quality Assessment Tool for Quantitative Studies* developed by the Effective Public Health Practice Project from the Ontario Ministry of Health in Canada [42], and recommended by other authors [43,44]. Studies were ranked as high, moderate or low in quality.

Disagreement regarding any article inclusion or exclusion was resolved through consultation with two authors with expertise in acculturation research (Renzaho, A & Swinburn, B). Due to the small number of studies retained (*n* = 9), a quantitative weighting by study size or quality (meta-analysis) was not conducted. Instead the strengths and limitations of each study were taken into account qualitatively in a synthesis of overall findings.

## Results

### Study selection

**Figure 1:**
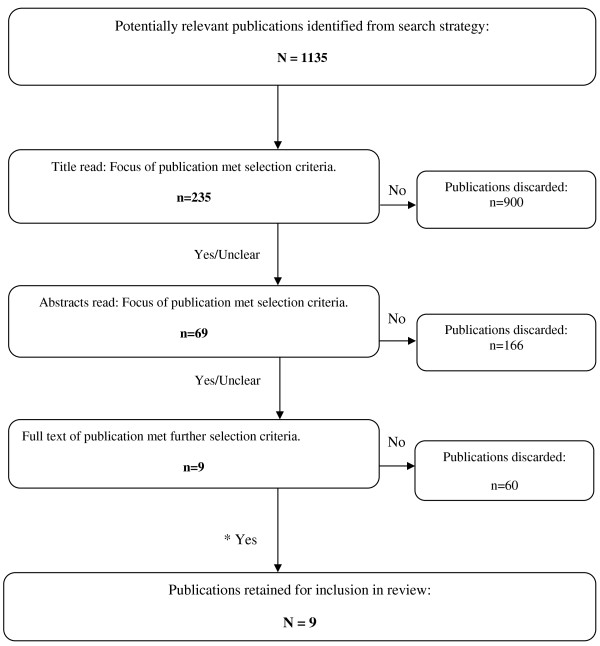
Process of study selection.

A total of 1135 papers were identified in the initial search. The vast majority of these were rejected on the basis of one or more of the following factors: the paper focussed only on acculturation, immigration, overweight or obesity rather than any relationship between the variables; the paper did not use a standardized (UDM or BDM) scale to measure acculturation; the paper did not include original, empirical research (commentaries, book reviews, ‘grey’ literature, etc.); or a combination of the above (see Figure 1). Overall, nine papers met the inclusion criteria (see Table 2).

**Table 2:** Studies included in systematic review

**Author (year)**	**Host country**	**Country of origin ( *N *)/ Gender**	**Study design**	**Acculturation scale**	**Measure of overweight /obesity**	**Results**	**Quality**	**Limitations**
Ahluwalia et al., [45]	USA	Mexico (1301, female: 664 male: 637)	Cross-sectional	Bi-dimensional acculturation scale (BDM)	Measured BMI, waist circumference	Higher degree of acculturation correlated with higher BMI. Among participants with a BMI ≥ 25, lower scores on acculturation predicted less likelihood of considering self as overweight (60% vs. 73%) and less likely of having attempted to lose weight (OR 5 0.49; 95% CI: 0.31–0.79).	Moderate	Certain sample subgroups were too small for empirical examination. Despite using the BDM acculturation model, the analysis treated as a UDM.
Barcenas et al., [46]	USA	Mexico (7503, female: 5471, male: 2032)	Cross-sectional	Bi-dimensional Acculturation Scale for Hispanics (BDM)	Self-reported BMI	Higher degree of acculturation and length of residency in the US were correlated with higher BMI: 46% of highly acculturated participants were obese compared with 43% with a low degree of acculturation (*p* < .0096).	High	Self-reported BMI cannot be verified and may bias results.
Bertera et al., [47]	USA	El Salvador (1205, female: 581 male: 624)	Cross-sectional	Uni-dimensional acculturation scale (UDM)	Measured BMI	Higher degree of acculturation was associated with higher BMI. As acculturation increased so too did BMI, and together with other variables accounted for 45% of the variance in BMI (*p* < .01-.001).	Moderate	Self-reported BMI cannot be verified and may bias results.
Fitzgerald et al., [48]	USA	Puerto Rico (200 female)	Cross-sectional	Uni-dimensional acculturation scale (UDM)	Measured BMI	Higher degree of acculturation was associated with higher BMI. Acculturation correlated positively with obesity; with less acculturated participants were 54% less likely to be obese.	Moderate	Data collection methods were not adequately reported.
Franzen et al., [49]	USA	Thailand/Laos, Hmong (65, female: 48, male: 17)	Cross-sectional	Short Acculturation Scale for Hispanics (BDM)	Measured BMI	Higher degree of acculturation was associated with higher BMI. Changes in the environment and increased acculturation level had negative effects on body weight and overall health.	Moderate	Participant attrition was not adequately reported.
Hazuda et al., [50]	USA	Mexico (2941, female: 1677, male: 1264)	Cross-sectional	Functional Integration with Mainstream Society scale (FIMS-scale), Value Placed on Preserving Mexican Cultural Origin scale (VPPMCO-scale) (BDM)	Measured BMI, sub scapular to triceps skin-fold thickness ratio, waist-to-hip circumference ratio.	In men, higher degree of acculturation was associated with higher BMI and less favourable body fat distribution. In women, higher degree of acculturation was associated with lower BMI and a more favourable body fat distribution (*p* = .01-.001). Prevalence of obesity was greater in Mexican-Americans than in non-Hispanic whites.	High	--
Khan et al., [51]	USA	Mexico (female: 1723, male 1418)-, Cuba (female: 451, male 377)-, and Puerto Rican- (female: 747, male: 64) (5180)	Cross-sectional	Uni-dimensional acculturation scale (UDM)	Measured BMI	Spanish language preference correlated positively with obesity, and acculturation measurement of generations correlated positively with obesity (*p* = .05-.001).	Moderate	The sample included second and third generation Cuban-Americans and Puerto Ricans as well as first generation migrants. For 2nd and 3rd generations, there was no full description of the process of acculturation for these subgroups of the sample. This may compromise comparability.
Lee et al., [52]	USA	Korea(356, female: ?, men: ?)	Cross-sectional	Two-culture Matrix Model based on Gordon’s theoretical work (BDM)	Self-reported BMI	Assimilated men recorded higher BMIs (1.7kg/m^2^ more) than men with lower degrees of acculturation (*p* < .05). Acculturation correlated positively with frequency of light physical activity (men OR = 4.34, *p* < .005; women OR = 7.38, *p* < .005) and better health in men (OR = 2.74, *p* < 05). No relationship between BMI and acculturation was present for women.	Moderate	Sample included second generation Korean Americans, and was generally of a higher SES than 1990 census.
Miller et al., [53]	USA	Soviet Union women (218)	Longitudinal (1 year duration)	American and Russian Behavioural Acculturation Scale (BDM)	Measured BMI & waist circumference	Maintaining origin cultural orientation correlated positively with higher waist circumference (β = −.15, *p* < .05) and BMI (*r*^*2*^ = .25, *p* < .01.	High	--

### Study characteristics & samples

All of the studies were published in the 1990s and 2000s, and all were conducted in the US. Eight studies utilised cross-sectional designs, while one was longitudinal (see Table 2). Of the studies reviewed, participant mean age was 41 years (SD = 15), and the male: female ratio across all studies was 32:68. The focus of the articles included was limited to immigrant and refugee resident populations, and thus, temporary residents were not represented in any of the studied populations. Study sample ethnicity included, Mexican-Americans [45,46,50,51], Hmong (Thailand and Laos) US residents [49]; Korean-Americans [52], Puerto-Rican Americans [48,51], and Soviet-American women [53]. Six studies used BDM-scales to measure acculturation [45,46,49,50,52,53] and three studies used UDM-scales [47,48,51]. Two studies used self-report measures for the BMI measure [46,52].

### Study quality

Table 2 presents the nine papers’ qualitative evaluations using Quality Assessment Tool for Quantitative Studies [46,50,53]. Three of the nine studies were classified as a ‘strong’ quality ranking while the remaining six were of ‘moderate’ quality [45,47-49,51,52]. Studies moderate in quality were so determined due to small sample sizes, data collection weaknesses, participant attrition and participant representation.

### Acculturation scales

The acculturation scales used in the reviewed studies are presented in Table 2. The scales were valid and reliable, and all comprised items gauging degree of acculturation/enculturation by typically focusing on language use, use of media in host country, ethnic social relations, social networks, lifestyle, values and/or attitudes. The studies employed both UDM and BDM scales, making it difficult to compare specific results across these studies using a common metric. However, the standardization, validity and reliability of the scales used, ultimately facilitated comparability and synthesis of the results obtained with each type of scale.

### Study findings

Of the nine studies reviewed, seven reported overall positive associations between the degree of acculturation and body weight variables [45-50,52]. The conclusions drawn from these studies generally complemented one another. Three studies [50,51,53] found negative associations between higher degree of acculturation and obesity in women. Lee et al. [52] found no significant relationship between acculturation and obesity in their female sub-sample, while more acculturated males recorded higher BMI.

In their study on US-born and Mexico-born Mexican-American adults living in the US, Barcenas et al. [46] indicated significant differences in the relationships between acculturation level and BMI, gender, and birthplace. In Mexican-born participants longer residency in the US was associated with higher BMI. Women residing in the US for more than 15 years recorded a mean BMI of 2.38 kg/m^2^ (*β* (adjusted) =2.12, 95% CI, 1.53-2.72, *p* < .0001) higher than those who had lived in the US for less than five years. Similarly, men living in the US for more than 15 years recorded an average BMI of 1.10 kg/m^2^ (*β* (adjusted) = 1.47, 95% CI, 0.59-2.34, *p* < .001) higher than residents of less than five years. Furthermore, the risk of obesity in men and women with a low degree of acculturation increased with every additional year of residence in the US by 2% (OR = 1.02, 95% CI, 1.00-1.03) and 1% (OR = 1.01, 95% CI, 1.01-1.02) respectively. For highly acculturated men and women the increase in risk was 4% (OR = 1.04, 95%CI, 1.01-1.07) and 3% (OR = 1.03, 95% CI, 1.00-1.05) respectively. In US-born Mexican-Americans, high acculturation and birthplace accounted for 6% and 25% of risk of obesity in men and women respectively.

These general findings of positive correlations between obesity and acculturation in Mexican-Americans, were further supported in the study by Ahluwalia et al., [45] where comparable general associations between the degree of acculturation and BMI were established. Further, Khan et al. [51] found that 2nd and 3rd generation Mexican-Americans had higher BMIs than 1st generation migrants.

A similar study was conducted by Hazuda et al. [50] who established a more ambiguous and complex relationship between acculturation and obesity in Mexican-Americans. In this study, overweight was assessed using BMI, sub scapular-to-triceps skin-fold thickness (SUBTR) ratio, and waist-to-hip ratio (WHR). Both acculturation and socio-economic status (SES) were associated with anthropometry measurements. Specifically, low SES was associated with a high WHR (*p* < .001), and high scores on the ‘family attitude’ subscale of acculturation was associated with a high SUBTR-ratio (*p* < 0.01). Among females, low SES was associated with high BMI, SUBTR-ratio and WHR (*p* < .01 – *p* < .001), but, interestingly, high scores on the ‘functional integration’ acculturation subscale were associated with lower BMI and SUBTR-ratios (*p* < .01 – *p* < .0001). This apparently protective effect for Mexican-American women was also evident in Khan et al. [51], where higher acculturation was associated with lower BMI for Mexican-American women (*β* = −.56, *p* < .01) but not for males nor either gender in the other ethnicities examined (Puerto Rican and Cuban).

Other studies examined the impact of acculturation on health factors such as smoking, physical activity, fat intake, and BMI, and have included other ethnicities with similar results. For example, Bertera et al. [47] found that acculturation was positively associated with obesity in Salvadoran immigrants in the US, accounting (in combination with other variables such as gender and SES) for 45% of the variance in immigrant obesity (*p* < .01-.001). Similarly, Fitzgerald et al. [48] discovered that less acculturated Puerto Ricans in the US were 54% less likely to be obese than their more acculturated counterparts. In yet other research on Hmong-American immigrants, higher degree of acculturation also correlated with higher BMI [49]. This relationship was mainly attributed to the changes in diet and physical activity following the adaptation to an environment with higher average income [49].

Comparable effects have also been found in studies among Korean-Americans [52] whereby more acculturated men (but not women) had a higher BMI than their enculturated counterparts, with a mean difference in BMI of 1.7kg/m^2^ (*p* < .05) [52]. This effect persisted when controlling for age, income, education, working status, and smoking as well as physical activity, fat intake and parents’ body size. In addition, and perhaps paradoxically, acculturated men and women were more likely to engage in light physical activity than enculturated men and women (men OR = 4.34, *p* < .005; women OR = 7.38, *p* < .005) [52].

The final paper included in this review [53] reported on a longitudinal study which examined the relationship between acculturation and cardiovascular disease risk factors in Soviet Union-born middle-aged women in the US. Similar to some of the other research reviewed, this study established a significant inverse correlation between acculturation score and obesity in women. Specifically, women with higher acculturation scores at baseline predicted lower changes in BMI over the following ten years (*r* = −.14, *p* = .05). Similarly, higher enculturation scores predicted higher changes in mean BMI and waist circumference (*r* = .25, *p* = .01; *r* = .34, *p* = .01, respectively) in the following decade [53].

Five of the nine studies included measurement of SES [47,48,50-52]. Three found no significant correlations between SES and obesity [47,51,52], while Fitzgerald et al. [48] established an inverse correlation between these two variables, and Hazuda et al. [50] found both positive and negative correlations for men and women, respectively.

## Discussion

The focus of this paper was to review the research among migrants on the relationship between obesity and acculturation/enculturation as measured by valid and comparable acculturation scales (uni-dimensional and bi-dimensional scales). Only nine studies met the criteria of this review, and all of them were US-based. With the exception of Miller et al. [53] and Khan et al. [51], all of the studies reviewed reported generally similar findings indicating an overall positive correlation between acculturation and overweight/obesity. Barcenas et al. [46] and Ahluwalia et al. [45] established positive correlations between acculturation and obesity in Mexican-Americans. The more acculturated the populations were, the higher the average BMI. While Khan et al. [51] found a negative relationship between BMI and acculturation in first-generation migrants, Mexican-Americans’ BMI increased with each generation in the US. Positive relationships between acculturation and obesity were also evident in the studies by Bertera et al. [47], Fitzgerald et al. [48], Franzen et al. [49] and Lee et al. [52] spanning a wide array of ethnicities (El Salvador, Puerto Rican, Hmong, Korean respectively). However, the relationship appeared more complex for women than for men, as both negative and positive relationships between acculturation and obesity were found for women [50,51,53].

These findings are likely to also be related to the *nutritional transition* documented in developing countries [54,55]. Nutritional transition relates to the tendency of decreased consumption of healthy and nutritional foods in favour of fatty and processed foods, observed among citizens in rapidly developing countries [55]. This effect has been detected globally as unhealthy processed foods are increasingly made available throughout the world [55]. A good example of this transition is Brazil where, since the 1970s, the problems of dietary deficiency have been replaced by those of dietary excess [56]. This nutrition transition would occur more rapidly in a migrating population as they make the transition over months and years as opposed to a whole country making the transition over years and decades. The nutrition transition for immigrants thus involves both the qualitative change in diet and the rapidity of that change which would be accentuated in migrants from low- and middle-income countries to high-income countries [55,56].

Looking beyond the overall tendencies in the relationship between obesity and acculturation, however, the relationship becomes more complex and appears to vary by gender, country of origin and SES. In some cases acculturation even seems to predict positive behaviour such as increased physical activity [46,52] and decreased CVD-risk and waist-circumference [53]. Specifically, Khan et al. [51], and Hazuda et al. [50], found protective effects of acculturation in terms of female body weight – that is, an inverse correlation between female BMI and acculturation was apparent in these studies in spite of the overall positive association between BMI and acculturation. Similar findings were evident in Miller et al. [53] study on female Soviet Union immigrants to the US. Of further relevance in this context, it is important to note that while Lee et al. [52] observed positive correlations between acculturation and body weight, this association held true only for men and not for women. Together, these studies thus lend credence to the idea of gender as a moderating variable in the relationship between acculturation/enculturation and obesity.

Despite the small number of publications, potential explanatory hypotheses can be developed for these emerging patterns. For the females in some migrating populations, it may be that as they get wealthier, the more health conscious they become, which in turn is reflected in behaviour and BMI. Such an effect has been documented for the relationship between SES and BMI where this association is positive until a certain level of wealth is reached after which the correlation inverts [57]. This flip in the relationship is much stronger for women than for men, and thus could account for the variability in results concerning female subgroups discussed in this review [57,58].

The counteracting environments and social norms in Western countries may have a differential impact on the migrating population depending on migrant country of origin, gender and SES [59,60]. The recurring finding that women with higher levels of acculturation have lower BMIs suggests that greater availability of fatty and processed foods in high income countries may be offset by the Western social norms and fashions which endorse fitness, health and a slim body shape [61-63]. This is supported in the study by Ahluwalia et al. [45] where it was established that obese Mexican-Americans with a low degree of acculturation were more satisfied with their weight and body shape, and less likely to have tried to lose weight than their highly acculturated counterparts. Further, Barcenas et al. [46] and Lee, et al. [52] found that in spite of a comparatively higher likelihood of increased BMI in acculturated men and women, this cohort was also more likely to engage in physical exercise than non-acculturated participants. These findings appear to highlight a possible association between acculturation and both adaptive and maladaptive health behaviours endorsing health ideals, such as physical activity and slenderness, *as well as* facilitating obesogenic habits.

### Strengths and limitations

A strength of this review relates to the chief goal of the paper which was to identify and review the research which has used valid and comparable acculturation scales to measure the relationship between acculturation and obesity in migrants to high-income countries. While only nine studies met the inclusion criteria for this paper, the fact that all of the reviewed research employed acculturation scales in their methodology effectively facilitated comparison and synthesis of the main themes and variations emanating from this research. This has not been done before and has thus far represented a gap in the literature on this topic. Nonetheless, although the studies included in our review used validated acculturation scales they varied in terms of number of items and domains covered. For example, some studies [45-48,51] included one dimension that focused on language acculturation rather than a broader array of behaviours related to the migration and settlement process. Others included questions regarding language use and preference, social connections, and overall eating patterns [49], while others still focused on functional integration (i.e. adoption of the values, attitudes, and behaviour of the host society) [50], structural acculturation [52], and language, identity, and behaviour [53]. In addition, when examining the relationship between acculturation and obesity, some studies used self-reported BMI measures whereas other used measured BMI, making it difficult to draw conclusions across studies. Thus, the variation in acculturation domains and measurement identified in this review emphasises the urgent need for a standardized international acculturation scale.

Another strength of this review centres on the broader research methodology of the included studies. Eight of them employed a cross-sectional design. While the correlational nature of this methodology prohibits a statistical assessment of cause and effect, it can be more readily applied to large representative samples as well as take into account a number of potentially confounding factors. Further, given the specific nature of the variables measured in the reviewed studies, it is still possible to hypothesise with relative certainty on the direction of the associations found despite the nature of cross-sectional research designs. That is, it is much more likely that acculturation has an impact on body weight than vice versa, and while several other factors may mediate or moderate the relationship, this direction of the association remains the most plausible.

A major limitation to the review, relates to the fact that all of the studies focussed on immigrants in the US – a country which is among the ten most obese nations in the world [64]. In light of this, it is conceivable that obesity may not be related to acculturation in general, but rather to the specific culture to which immigrants acculturate. That is, immigration and acculturation to a country where obesity is not a prominent health issue may not increase migrant BMI. More research examining immigration to countries other than the US is therefore needed for more generalisable conclusions.

Another limitation relates to acculturation scales used. The introduction to this study identified the bi-directional model of acculturation as being more complex and sophisticated than the uni-dimensional model. The current review included studies which used both types of scales, making it difficult to compare specific results across these studies using a common metric. There is also some debate focusing on socioeconomic status and its possible role on the relationship between acculturation and obesity. However, not all the nine studies took this into account and therefore lack of adequate control for SES perhaps partly explain the distinct findings among women.

Finally, the review included studies which used both measured and self-reported BMI (Barcenas et al., 2007; Lee et al., 2000). This comprises a limitation as the accuracy of self-reported BMIs may be questionable.

## Conclusions

This review is the first of its kind focussing solely on migrant obesity studies using either bi-directional or uni-directional measurements of acculturation rather than utilizing a surrogate measurement.

Despite the different but standardized measures of acculturation in the studies reviewed here, the findings seem to be relatively consistent. This uniformity has not been observed in studies that solely used a proxy measure for acculturation. It therefore seems imperative to endorse the use of standardized scales of acculturation in future research.

The degree of acculturation seems to reflect the degree of nutrition transition towards a more obesogenic diet and higher BMI. However, there appears also to be some countervailing forces of acculturation that precipitate a more complex pattern for women. There was some evidence of higher physical activity levels among women. Leaner body image ideals and variable SES in high-income countries may explain the mixed findings for the relationship between acculturation and female BMI. Future research should help further unpack this interchange of culture, migration, gender and SES on the development of obesity. Identifying underlying contributors of diet, physical activity and body size perceptions will also be important for this understanding and for informing potential interventions to prevent unhealthy weight gain.

Further, future research should examine the protective factors of enculturation with a focus on retaining the positive parts of the traditional orientation to combine with the positive aspects of the host culture. High income countries (such as the US, Australia and many European countries) which have substantial migrant population from low- and middle-income countries should be active in their efforts to prevent obesity and obesity-related diseases like diabetes in these at-risk populations.

### Key messages

•Based on the reviewed studies, there appears to be an overall positive relationship between acculturation and obesity in populations migrating to high-income countries from low- to middle-income countries.

•Migrant gender appears to be a significant factor in the relationship between acculturation and obesity. Due to the limited valid research on this topic, future study should focus on this relationship.

•The role of nutrition transition in the relationship between acculturation and obesity also is likely to play an important role in the connection between acculturation and migrant weight gain and obesity.

•For the sake of empirical reliability and rigour the use of validated, comprehensive and uniform acculturation scales should feature prominently in future study.

### Endnote

^1^The authors define high, middle and low income countries based on United Nation criteria.

## Competing interests

The authors declare that they have no competing interests.

## Authors’ contributions

All authors have made substantive intellectual contributions to this study. 1) MD and AR have made substantial contributions to conception and design. 2) MD, ALS and AR carried out acquisition of data. 3) MD, ALS, BS and DM performed analysis and helped to interpret the data. 4) MD, ALS, BS and DM have drafted the manuscript and revised it critically for important intellectual content. 5) MD, ALS, BS and DM read and approved the final manuscript. 6) All authors have given final approval of the version to be published.

## Authors’ information

MD is a PhD Scholar in WHO Collaborating Centre for Obesity Prevention, Department of Health& Social development, Deakin University, Australia.

ALS is a PhD Scholar in the Department of Psychology, College of Life and Environmental Sciences, University of Exeter, UK.

BS is a Professor of Population Nutrition and Global Health at University of Auckland as well as Alfred Deakin Professor and Director of WHO Collaborating Centre for Obesity Prevention at Deakin University, Australia.

DM is a Professor in the School of Psychology and Associate Director of the Centre for Mental Health and Wellbeing Research at Deakin University, Australia.

AR is the Director of the Migration, Social Disadvantage, and Health Programs in Department of Epidemiology and Preventive Medicine, Monash University, Australia.

## Pre-publication history

The pre-publication history for this paper can be accessed here:

http://www.biomedcentral.com/1471-2458/13/458/prepub
